# Social Capital Enhanced Disaster Preparedness and Health Consultations after the 2011 Great East Japan Earthquake and Nuclear Power Station Accident

**DOI:** 10.3390/ijerph15030516

**Published:** 2018-03-14

**Authors:** Makoto Hasegawa, Michio Murakami, Yoshitake Takebayashi, Satoshi Suzuki, Hitoshi Ohto

**Affiliations:** 1 Department of Health Risk Communication, Fukushima Medical University, 1 Hikarigaoka, Fukushima City, Fukushima 960-1295, Japan; m131094@fmu.ac.jp (M.H.); ytake2@fmu.ac.jp (Y.T.); 2 Fukushima Prefectural Centre for Environmental Creation, 2-10 Fukasaku, Miharu-machi, Tamura-gun, Fukushima 963-7700, Japan; suzuki_satoshi_03@pref.fukushima.lg.jp; 3 Department of Blood Transfusion and Transplantation Immunology, Fukushima Medical University, 1 Hikarigaoka, Fukushima City, Fukushima 960-1295, Japan; hit-ohto@fmu.ac.jp; 4 Department of Advanced Cancer Immunotherapy, Fukushima Medical University, 1 Hikarigaoka, Fukushima City, Fukushima 960-1295, Japan

**Keywords:** disaster preparedness, disaster risk reduction, Fukushima Daiichi Nuclear Power Station, Great East Japan Earthquake, healthcare checkups, social capital

## Abstract

After the Great East Japan Earthquake and the subsequent Fukushima Daiichi Nuclear Power Station accident in 2011, there was a strong demand to promote disaster preparedness approaches and health checkups for the prevention of lifestyle diseases. This study examined the yearly change in the percentage of those who prepared for disasters and who utilized health checkups in Fukushima Prefecture, and identified the factors governing disaster preparedness and utilization of health checkups. We used the public opinion survey from 2011 to 2015 (*n* = 677–779 each year) on prefectural policies that is conducted every year by the Fukushima Prefecture government Public Consultation Unit. We found that the percentage of those who prepare for disasters decreased, while that for health checkups did not significantly change. With regard to disaster preparedness, experiences of disaster enhance disaster preparedness, while bonds with other local people help to maintain preparedness. For health checkups, familiarity with the welfare service was the most important factor governing such consultations. The findings suggest that social capital should be promoted in order to improve disaster preparedness. The findings also suggest that residents’ accessibility to medical and welfare services is also important in promoting the utilization of health checkups.

## 1. Introduction

The Great East Japan Earthquake and the subsequent accident at Tokyo Electric Power Company’s Fukushima Daiichi Nuclear Power Station in 2011 (hereinafter, we call both of them the 2011 disaster as a whole) caused extensive damage in Fukushima Prefecture and other surrounding regions. Due to a lack of food following the 2011 disaster, residents relied mostly on private supplies [1], illustrating the importance of building stockpiles and confirming evacuation sites to prepare for future disasters. Radiation exposure was well controlled and minimal [2], but lifestyle diseases such as diabetes and hyperlipidemia emerged as a major health issue in the affected people [3,4,5,6]. Based on lessons learned from the 2011 disaster, there is a need to prevent diseases, promote health, and improve early detection of disease, especially among evacuees. Furthermore, following the 2011 disaster, aging and population decline also became serious issues in Evacuation Order Areas (EOAs) [7], which increases a concern of possible weakened community resilience or social capital (e.g., social networks, reciprocal ties, social participation [8,9]). The Sendai Framework was adopted by the 187 member states, highlighting the importance of social resilience toward disaster risk reduction [10].

Several previous studies have investigated factors associated with disaster preparedness and personal health checkups. For example, Ozkazanc and Yuksel [11] investigated highly educated students in Turkey. They found that while 55.1% of students had experienced one or more disaster incident, 78.6% had not received any training courses related to disasters during their time in formal education; 95.2% did not own disaster and emergency situation kits; and 82.9% were unaware of the designated safe zones within their residences. In a similar study on disaster preparedness, Hoffmann and Muttarak [9] showed that high social capital, in addition to education and disaster experiences, promotes disaster preparedness. In 2016 the New Zealand Government’s Ministry of Civil Defence and Emergency Management reported that the sense of urgency brought about by the 2010–2011 Canterbury earthquakes, which propelled people into preparing, had temporally worn off [12]. When it comes to health services, several factors impact utilization [13,14,15,16]. For instance, advanced age impacts willingness to bear out-of-pocket checkup expenses [14], while lack of money, time, and related knowledge prevents people from utilizing cancer screening [15]. Nagai et al. reported that changes of socioeconomic status and community activities were associated with subjective perceptions of health [17].

Although several studies have investigated either disaster preparedness or health checkup utilization, none have yet comprehensively analyzed factors of both disaster preparedness and post- disaster health checkups together. It is expected that identifying the factors that govern in both domains, and clarifying commonalities and differences between the two, will enhance understanding of how to promote disaster preparedness and post-disaster wellbeing. As described above, social capital possibly enhances both disaster preparedness and health checkup utilization. Furthermore, no Japanese study to date has identified factors pertaining to disaster preparedness and health checkups specifically following the 2011 disaster, and their yearly changes. Nakayachi and Nagaya’s study reported that Japanese people had more anxiety about an earthquake one year after the 2011 disaster, while four years later their anxiety had declined to the same level as before the 2011 disaster [18]. Thus, disaster preparedness may exhibit a similar decline. It is therefore necessary to evaluate whether people who prepare for disasters exhibited less inclination to do so in the years following the 2011 disaster, and, if so, to identify the factors that impacted this decline. It is also considered beneficial for developing health policy to identify factors that encourage people to pursue health checkups in situations in which a decline in health conditions is reported—specifically, the aftermath of the 2011 disaster.

This paper has two objectives: first, we clarified the yearly changes of those who prepared for disasters and who utilized health checkups in Fukushima Prefecture. Second, we identified the factors governing disaster preparedness and health checkups, and investigate their changes over the years. Following previous studies described above, we hypothesized that social capital was associated with both disaster preparedness and health checkups. Furthermore, we investigated factors such as evacuation experiences, in addition to demographic parameters such as sex and age, relief (low anxiety) regarding disasters, and familiarity with medical and caregiving services. This is the first study to identify factors governing disaster preparedness and health checkups, and the yearly changes following a disaster.

## 2. Materials and Methods

### 2.1. Questionnaires

**Figure 1:**
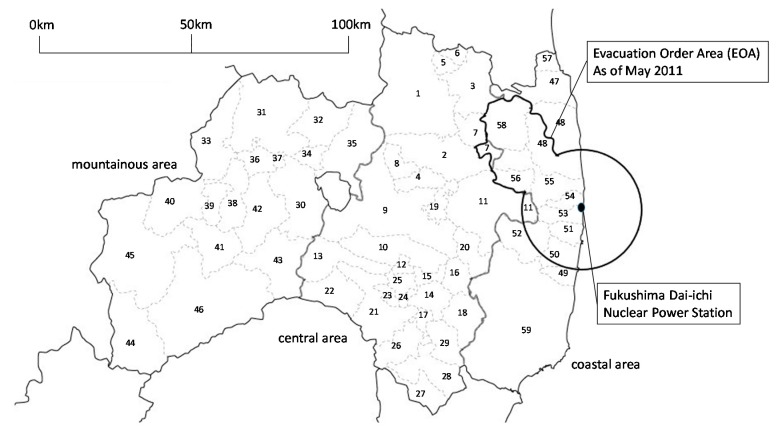
Map of municipalities in Fukushima Prefecture. EOA = evacuation order areas. Central area: 1. Fukushima City, 2. Nihonmatsu City, 3. Date city, 4. Motomiya City, 5. Ko-ori Town, 6. Kunimi Town, 7. Kawamata Town, 8. Otama Town, 9. Koriyama City, 10. Sukagawa City, 11. Tamura City, 12. Kagamiishi Town, 13. Tenei Village, 14. Ishikawa Town, 15. Tamakawa Village, 16. Hirata Village, 17. Asakawa Town, 18. Furudono Town, 19. Miharu Town, 20. Ono Town, 21. Shirakawa Town, 22. Nishigou Village, 23. Izumizaki Village, 24. Nakajima Village, 25. Yabuki Village, 26. Tanagura Village, 27. Yamatsuri Town, 28. Hanawa Town, 29. Samegawa Village. Mountainous area, 30. Aizuwakamatsu City, 31. Kitakata City, 32. Kitashiobara Village, 33. Nishiaizu Town, 34. Bandai Town, 35. Inawashiro Town, 36. Aizubange Town, 37. Yugawa Village, 38. Yanaizu Town, 39. Mishima Town, 40. Kaneyama Town, 41. Showa Village, 42. Aizumisato Town, 43. Shimogo Town, 44. Hinoemata Village, 45. Tadami Town, 46. Minami-aizu Town. Coastal area other than EOAs, 47. Soma City, 57. Shinchi Town, 59. Iwaki City. EOAs, 48. Minamisoma City, 49. Hirono Town, 50. Naraha Town, 51. Tomioka Town, 52. Kawauchi Village, 53. Okuma Town, 54. Futaba Town, 55. Namie Town, 56. Katsurao Village, 58. Iitate Village. Survey area for each year. 2010: 1–5, 8–12, 15, 19, 21, 22, 28–31, 35, 38, 42, 46–48, 51, 54, 57, 59. 2011: 1–5, 7, 9–12, 14, 16, 21, 23, 25, 26, 36, 40, 42, 43, 47, 48, 50–52, 59. 2012: 1–4, 6, 8–11, 13, 17, 19, 21, 22, 25, 30, 31, 34, 36, 42, 45, 47, 48, 51, 53, 57, 59. 2013: 1–5, 7, 9–12, 18, 20, 21, 23, 26, 27, 30, 31, 33, 38, 42, 46–49, 51, 52, 59. 2014: 1–4, 6, 8–11, 14, 18, 20–22, 24, 26, 30, 31, 33, 37, 42, 46–49, 52, 55, 59. 2015: 1–5, 7, 9–12, 15, 19, 21, 22, 25, 26, 30, 31, 34, 36, 42, 46–48, 51, 54, 57, 59.

We obtained permission from the Fukushima Prefectural government to use the data from the annual public opinion survey on prefectural policies that is conducted by the Fukushima Prefecture government Public Consultation Unit. We used surveys from 2011 to 2015 for the study. The subjects comprise males and females aged 15 years or older, sampled from selected cities, towns, and villages in the prefecture using a stratified two-stage random sampling method (Figure 1). Since the questionnaires were sent to people based on the Basic Resident Register, these forms were also sent to those who had evacuated from EOAs via forwarding services. Although population declines have occurred especially in EOAs, the whole population in Fukushima Prefecture did not change significantly between 2010 (2,029,064 people) and 2015 (1,914,039) [19]. The people who received the survey changed each year [20]. The questionnaires are sent via mail to 1300 different respondents each year [20]. In this study, we only analyzed respondents aged 20 or older, and 677–779 respondents within this category were targeted each year (Table 1).

For disaster preparedness (A1), we used the questionnaire item “Do you prepare for large-scale disasters (e.g., to ensure that you are aware of the nearest evacuation site, and to build stockpiles)?” For health checkups (A2), we used “Do you utilize health checkups to assess your risk of lifestyle diseases, and so on?” Five answer choices were provided: “yes,” “somewhat yes,” “neither yes nor no/not applicable,” “somewhat no,” and “no.” The question items related to disaster preparedness and health checkups were asked from 2011 and 2012, respectively.

Furthermore, we used the respondents’ age, sex, region of residence, relief (low anxiety) level regarding regional disasters and radiation, and presence or absence of medical and welfare services in the region, along with the overall community awareness level on reconstruction and social bonds (see details below). The overall community awareness level was used to reflect social capital status within the area. Living region for evacuees refers to living region before the 2011 accident.

Regarding relief level, we used risk perceptions related to regional disasters (Q1) and radiation (Q2). Relief regarding regional disasters was assessed using the following: “Is the region you live in resistant to and safe from disasters such as flood, earthquake, and fire?” in 2011 and 2012, and “Is the region you live well resistant and secure against natural disasters and large-scale fire?” in 2013, 2014, and 2015. Although the two items used differ, we regard this difference as negligible. For relief regarding radiation, we used a following item: “Is your living space secure against radiation?”

Regarding presence or absence of medical and welfare services, we used questionnaire items related to participants’ familiarity with such services, including: “Does your place of residence have essential medical facilities in the immediate vicinity?” (Q3), and “Does your place of residence have essential welfare services in your back yard?” (Q4).

Regarding overall community awareness level, we used questionnaire items to evaluate areas pertaining to prefectural reconstruction and bonds between survey participants and other local people, as follows: “Do you think Fukushima Prefecture has made sufficient effort to reconstruct areas affected by the nuclear disaster?” (Q5) and “Do you experience mutual support and bonds with other local people on a day-to-day basis?” (Q6). Questionnaire items Q2–Q6 have been asked since 2012. Five answer choices were provided for items Q1–Q6, similar to those outlined above.

The Fukushima Medical University Ethics Committee (2899) approved this study and gave permission for the use of their data.

### 2.2. Statistical Analysis

For the analysis, we classified the data as follows. For personal attributes, age was classified into five groups (20–29, 30–39, 40–49, 50–59, 60 or over), and region of residence into four groups (mountainous area [Aizu]; central area [Nakadori]; coastal area [Hamadori] other than EOAs; and EOAs). The answers related to disaster preparedness (A1), health checkups (A2), and Q1–Q6 were classified into two groups: “yes” and “somewhat yes” were classified as “1” and “neither yes nor no/not applicable,” “somewhat no,” and “no” were classified as “0.” The references of explanatory variables were set as follows: female for sex, 20s for age, and mountainous area (the least affected region) for region of residence. This classification was consistent with our previous study using the public opinion survey conducted every year by the Fukushima Prefecture government Public Consultation Unit [21].

We used a chi-squired test to confirm the association between disaster preparedness (A1) and utilization of health checkups (A2) at first. Next, we used trend analysis to investigate the statistical change in the utilization of disaster preparedness (A1) and health checkups (A2) in individual regions. We then conducted a logistic regression analysis to identify the factors governing disaster preparedness (A1) and utilization of health checkups (A2) each year. Considering data availability, we analyzed five models. In Model 1, we analyzed the data from 2012 to 2015. The objective variables were those who had both disaster preparedness (A1) and utilization of health checkups (A2), and explanatory variables were age, sex, region, and Q1–Q6. In Model 2, we analyzed the data from 2011 to 2015. The objective variable was disaster preparedness (A1), and explanatory variables were age, sex, region, and Q1. In Model 3, we analyzed the data from 2012 to 2015. The objective variable was disaster preparedness (A1), and explanatory variables were age, sex, region, and Q1–Q6. In Model 4, we analyzed the data from 2012 to 2015. The objective variable was health checkups (A2), and explanatory variables were age, sex, region, and Q1–Q6. In Model 5, since people aged 50 and older tended to seek health checkups in Model 4, we divided the data by age into “under 50” and “50 or over,” and performed a logistic regression analysis similar to that applied to Model 4. Low multicollinearity (variance inflation factor 1.005–4.477) was confirmed in the analyses.

Since the logistic analysis showed an association between disaster preparedness (A1) and Q6, we divided the data according to the absence or presence of bonds with other locals to perform the trend analysis of yearly changes in disaster preparedness (A1). We also performed a trend analysis to investigate the yearly changes of proportion of people who had bonds with other locals.

IBM SPSS Statistics 24 (IBM, Armonk, NY, USA) was used in the analysis.

## 3. Results

**Figure 2:**
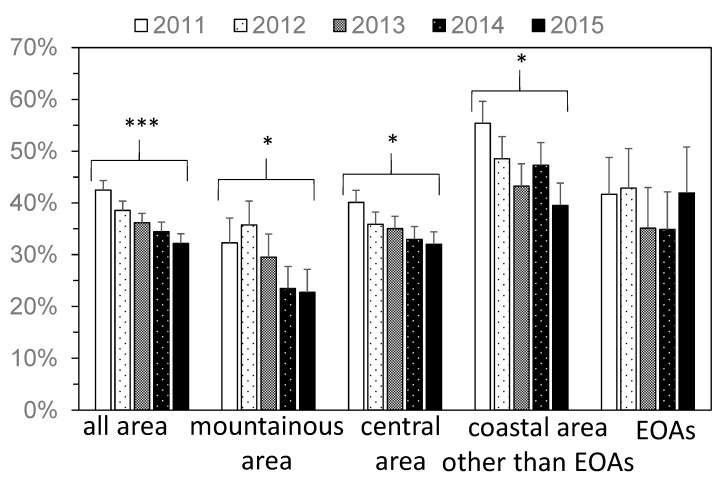
The yearly change in the percentage of people who prepared for disasters (A1). Error bar represents standard error. EOA = evacuation order areas. * *p* ≤ 0.05, *** *p* ≤ 0.001.

After the 2011 disaster, the percentage of people in Fukushima Prefecture who prepared for disasters significantly decreased with years (Figure 2): specifically, 42% of people prepared for disasters in 2011, but this value had decreased to 32% in 2015. There was no significant change in the EOAs; however, there were significant decreases in the mountainous area, central area, and coastal area other than EOAs. In particular, in the coastal area other than EOAs, the percentage significantly decreased, from 55% in 2011 to 40% in 2015.

**Figure 3:**
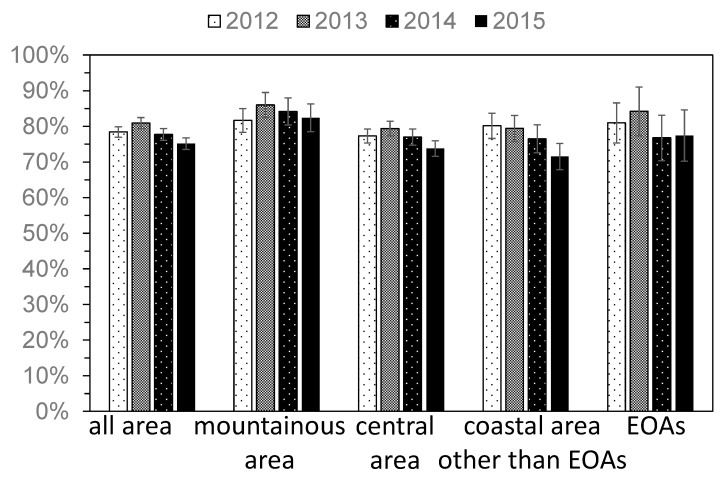
The yearly change in the percentage of people who utilized health checkups (A2). Error bar represents standard error. EOA = evacuation order areas.

On the other hand, the percentage of people who utilized health checkups did not change significantly in any region (Figure 3), at 78% in 2011 and 75% in 2015.

We found significant association between disaster preparedness (A1) and utilization of health checkups (A2) in 2013 and 2014 (Table 2). People sought health checkups regardless of disaster preparedness; however, people who had prepared for a disaster were more eager to utilize health checkups.

We conducted a logistic analysis to identify factors governing disaster preparedness (A1) and health checkups (A2) (Table 3, Table 4, Table 5, Table 6, Table 7 and Table 8). In Model 1, the percentages of people who had both disaster preparedness (A1) and health checkups (A2) were significantly higher in the coastal area other than EOAs compared to that in the mountainous area in 2014 and 2015. Q6 (bonds with other local people) was also significantly associated with the percentages of people who had both disaster preparedness (A1) and health checkups (A2) for four years.

In Model 2, disaster preparedness (A1) among men was continuously lower than that among women from 2011–2013, but it became similar from 2014 onward. A significant association was observed between regions and disaster preparedness (A1) in all years. In particular, disaster preparedness (A1) in the coastal area other than EOAs was significantly higher than that in the mountainous area. Similarly, disaster preparedness (A1) in the EOAs was significantly higher than that in the mountainous area in 2014, and we found a similar trend in 2015. Q1 (relief regarding regional disasters) was significantly associated with disaster preparedness (A1) in 2011, 2012, and 2015.

In Model 3, in addition to the results found in Model 2, Q6 (bonds with other local people) was significantly associated with disaster preparedness (A1) for 2012–2015.

In Model 4, we observed a significant association between age and the utilization of health checkups (A2) in 2012–2015. The utilization of health checkups (A2) for people in their 40s and 50s, as well as those who were older, was significantly higher than utilization for people 20–29 in 2013–2015 and in all years, respectively. We observed a significant association between the utilization of health checkups (A2) and bonds with other local people (Q6) in only 2013. In Model 5, we observed a significant association between familiarity with the welfare service (Q4) and health checkups (A2) in 2013 and 2014 for those under 50 only.

**Figure 4:**
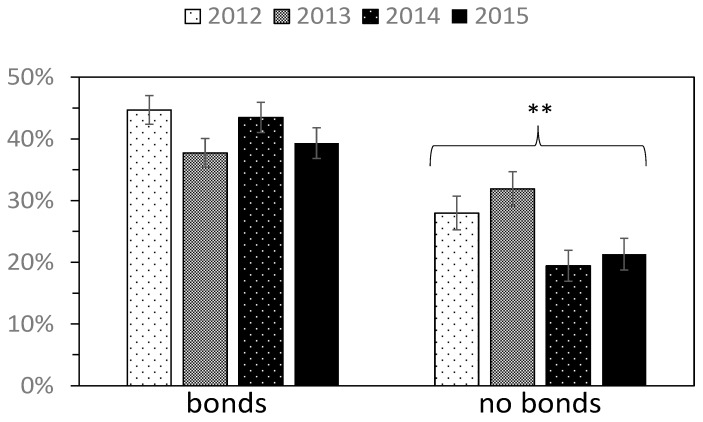
The yearly change in the percentage of people who prepared for disasters (A1) (respondents were divided into two categories according to the presence or absence of bonds with other local people). Error bar represents standard error. ** *p* ≤ 0.01. We separately conducted trend analysis to those who felt bonds with other local people and those who did not.

Following the results for Model 3, which showed a significant association between Q6 (bonds with other local people) and disaster preparedness (A1), we divided respondents into two groups on the basis of presence or absence of bonds with other locals, and then observed the yearly change in the percentage of people who prepared for disasters (A1) in each group (Figure 4). For those who did not experience bonds with other locals (Q6), the disaster preparedness showed a significant decline (from 28% in 2012 to 21% in 2015). However, for those who experienced bonds with other locals, disaster preparedness did not show a significant decline (from 45% in 2012 to 38% in 2015). The percentage of people who experienced bonds with other local people (Q6) did not significantly decrease, irrespective of region (Figure S1).

## 4. Discussion

We found an association between disaster preparedness (A1) and utilization of health checkups (A2). Furthermore, we found that people who reported stronger bonds with other local people (Q6) were more likely to engage in both disaster preparedness (A1) and utilization of health checkups (A2).

After the 2011 disaster, the percentage of people who prepared for disasters decreased in the Fukushima Prefecture. Thus, it seems that people tend to forget to prepare for disasters—even in areas affected by the 2011 disaster. This result is consistent with a 2016 finding by the New Zealand Government’s Ministry of Civil Defense and Emergency Management [12]. We found that sex, region, relief regarding regional disasters, and bonds with other local people were significantly associated with disaster preparedness (A1). Regarding sex, women prepared more for disasters compared to men, which is consistent with a finding by Hoffmann and Muttarak [9]. Although this difference between the sexes has been insignificant since 2014, the reason for the insignificance of the difference is not clear. Further studies are needed to identify the reason. For region, the percentage of those who prepared for disasters was higher in the coastal areas (i.e., the affected area) than in the mountainous area (i.e., the least affected area). This indicates that disaster experiences enhance disaster preparedness (A1), which is also in accordance with Hoffmann and Muttarak [9]. Although the percentage of those who prepared for disasters in the coastal area other than EOAs was significantly higher in all years compared to those in the mountainous area, we found significant differences for only one year in Model 2, and for two years in Model 3 in the EOAs. This may be attributed to two reasons. First, the number of respondents who lived in the EOAs was small, and the statistical power was therefore weak. Second, the evacuation changed people’s lives, especially just after the disaster, and therefore they may not have been able to afford to engage in disaster preparedness (A1). It may be difficult for evacuees to know the nearest evacuation site, which could be unfamiliar to them. This possibility is supported by the fact that a significant difference was found after 2014. The odds ratios (ORs) for EOAs were significantly higher than 1 only in the 2014 in Model 2 and in the 2014–2015 in Model 3. After 2014, people in EOAs might be able to afford to engage in disaster preparedness (A1). With respect to relief regarding regional disasters (Q1), we found that this was significantly and positively associated with disaster preparedness (A1). This represents that the more people think of their region of residence as resistant to and safe from disasters, the more likely they are to prepare for disasters. Relief regarding regional disasters (Q1) may enhance disaster preparedness (A1), although the mechanism is not clear. In this regard, however, this result might involve a contrary causal effect: the more people prepare for disasters, the more they think of their region of residence as disaster resistant and safe.

We identified bonds with other local people as another factor governing disaster preparedness (A1), observing positive associations between these factors all the years. Hoffmann and Muttarak mentioned that strong social capital promotes preparatory actions against disasters [9]. If we interpret “bonds” in this study as a proxy for social cooperation activities within the community, our results can suggest that social cooperation activities within the community are useful to enhance awareness of disaster preparedness (A1).

For health checkups (A2), we found a positive association between aging and the utilization of health checkups, which is consistent with findings by Lee et al. [14]. This can be attributed to combined effects stemming from the fact that specific health checkups (i.e., public health checkups) are target at those aged 40 or over, that aged retired people have more time to pursue health checkups [15], and that physical deterioration with age may increase interest in health.

Furthermore, we found a positive association between familiarity with welfare services (Q4) and health checkups (A2) among those aged under 50. Accessibility of such services was regarded as an important factor in health checkups (A2). This is consistent with the findings that accessibility to the examination with a whole body counter (i.e., an instrument to monitor internal radiation exposure) enhances participation [16].

Regarding to social capital, we could not observe a significant association between the utilization of health checkups (A2) and bonds with other local people (Q6) except 2013. People who had stronger bonds possibly tended to utilize more health checkups; however, people with low bonds were likely to have poor health status, facilitating the utilization of health checkups (A2). These two opposing factors might weaken the association between the utilization of health checkups (A2) and bonds with other local people (Q6).

However, bonds with other local people (Q6) were associated with people who had both disaster preparedness (A1) and health checkups (A2) all the years (Table 3). Thus, promoting social cooperation for isolated people is important to improve both disaster preparedness (A1) and health checkups (A2). Since strong social capital also had the advantage of reducing the risk of cognitive decline following the 2011 disaster [22], such policies would be beneficial.

We found uncommon factors as well (e.g., region, familiarity with the welfare service (Q4), and age). This suggests that different psychological and social factors also contribute to disaster preparedness (A1) and health checkups (A2). The experiences of the 2011 disaster improved only disaster preparedness (A1). Familiarity with welfare services (Q4) was suggested only to improve the utilization of health checkups (A2). Aging was also found to be associated with the utilization of health checkups (A2). Despite the particular increase in lifestyle diseases among residents in EOAs [3,4,5], an increase in health checkups was not observed among this group. Indeed, the rate of participation in specific health checkups in the Fukushima Prefecture did not increase compared to the national average [23]. To promote participation, it is important to prepare medical and welfare services for residents and to promote them in order to increase residents’ familiarity with them. Tanimura et al. reported that travel time and a type of medical department significantly affected the selection of health facilities in Benin [24]. Mahmud and Aljunid also reported the association between travel impedance and utilization for mammogram screening [25].

Despite the study’s pertinent findings, it also has some limitations. First, it was cross-sectional in nature, so there remains room to discuss causal effects. Second, some municipalities (e.g., Namie Town and Iitate Village) were selected at no time or for limited times during the survey periods (Figure 1); this selection might affect the yearly changes. Third, the small sample size resulted in low statistical power. Future studies should investigate the factors that were shown to be insignificant in this study (e.g., the association between EOAs and disaster preparedness). Furthermore, additional research is warranted to examine whether disaster preparedness (A1) and health checkups (A2) are promoted by policies and interventions such as improvements to social cooperative activities within communities, and accessibility to medical and welfare services.

## 5. Conclusions

After the 2011 disaster, the percentage of people who prepared for disasters decreased in Fukushima Prefecture, even in areas affected by the disaster. Bonds with other local people (Q6) were associated with people who had both disaster preparedness (A1) and health checkups (A2). The experiences of the 2011 disaster improved disaster preparedness (A1), and bonds with other local people (Q6) helped to maintain preparedness. Accessibility to such services is also important to promote the utilization of health checkups (A2).

## Figures and Tables

**Table 1:** Basic information on respondents. EOAs = evacuation order areas.

Year		2011	2012	2013	2014	2015
Valid respondents		779	749	760	705	677
(Ratio %)		(59.9%)	(57.6%)	(58.5%)	(54.2%)	(52.1%)
Sex	Men	319	324	332	280	286
	Women	454	422	421	421	388
	No response	6	3	7	4	3
Age	20s	72	46	48	46	39
	30s	97	110	84	87	74
	40s	115	102	102	89	93
	50s	163	144	128	125	112
	60 years and over	332	347	398	358	359
Region	Mountainous area	108	110	111	106	93
	Central area	463	428	435	385	385
	Coastal area other than EOAs	141	138	139	137	132
	EOAs	46	42	38	44	32
	No response and others	21	31	37	33	35

**Table 2:** The association between disaster preparedness (A1) & utilization of health checkups (A2). ns = *p* > 0.05, *** *p* ≤ 0.001.

		People Who Did Not Utilize Health Checkups	People Who Utilized Health Checkups	Odds Ratio	
2012	People who did not prepare for disasters	105	340	1.37	ns
	People who prepared for disasters	50	222		
2013	People who did not prepare for disasters	105	348	2.06	***
	People who prepared for disasters	33	225		
2014	People who did not prepare for disasters	116	315	2.34	***
	People who prepared for disasters	31	197		
2015	People who did not prepare for disasters	118	317	1.45	ns
	People who prepared for disasters	42	164		

**Table 3:** The associations between those who had both disaster preparedness (A1) and utilization of health checkups (A2) and factors (Model 1). CI = confidence interval. ref. = reference. EOA = evacuation order areas. ns = *p* > 0.05, * *p* ≤ 0.05, ** *p* ≤ 0.01, *** *p* ≤ 0.001.

	2011		2012		2013		2014		2015	
	Odds Ratio (95% CI)		Odds Ratio (95% CI)		Odds Ratio (95% CI)		Odds Ratio (95% CI)		Odds Ratio (95% CI)	
Model 1										
Women (ref)	-		1		1		1		1	
Men	-		0.73 (0.52–1.03)	ns	0.84 (0.59–1.20)	ns	0.92 (0.63–1.34)	ns	0.93 (0.62–1.40)	ns
20–29	-		1		1		1		1	
30–39	-		2.30 (0.95–5.55)	ns	1.67 (0.63–4.46)	ns	1.42 (0.58–3.49)	ns	1.00 (0.36–2.81)	ns
40–49	-		2.75 (1.13–6.71)	*	3.44 (1.36–8.73)	**	1.22(0.50–2.96)	ns	1.03 (0.39–2.72)	ns
50–59	-		1.68 (0.70–4.03)	ns	2.32 (0.93–5.82)	ns	1.09 (0.46–2.60)	ns	1.37 (0.54–3.48)	ns
60 or over	-		2.08 (0.92–4.71)	ns	2.74 (1.16–6.47)	*	1.85 (0.85–4.02)	ns	1.35 (0.57–3.17)	ns
Mountainous area (ref)	-		1		1		1		1	
Central area	-		1.03 (0.62–1.71)	ns	1.04 (0.61–1.79)	ns	1.94 (1.07–3.53)	*	1.40 (0.74–2.64)	*
Coastal area other than EOAs	-		1.58 (0.89–2.82)	ns	1.78 (0.95–3.33)	ns	3.63 (1.86–7.08)	***	2.45 (1.21–4.96)	***
EOAs	-		1.48 (0.65–3.34)	ns	1.77 (0.73–4.28)	ns	2.13 (0.85–5.31)	ns	2.30 (0.85–6.22)	ns
Q1 (relief regarding regional disasters)	-		1.40 (0.96–2.05)	ns	1.04 (0.71–1.53)	ns	0.88 (0.60–1.29)	ns	1.18 (0.78–1.78)	ns
Q2 (relief regarding of radiation)	-		0.93 (0.62–1.40)	ns	1.08 (0.72–1.62)	ns	1.35 (0.90–2.03)	ns	0.84 (0.55–1.29)	ns
Q3 (familiarity with the medical service)	-		1.03 (0.65–1.64)	ns	1.73 (1.05–2.85)	*	1.03 (0.62–1.72)	ns	1.25 (0.65–2.37)	ns
Q4 (familiarity with the welfare service)	-		1.19 (0.80–1.76)	ns	0.95 (0.62–1.45)	ns	1.46 (0.93–2.30)	ns	1.44 (0.86–2.41)	ns
Q5 (evaluation of prefectural reconstruction)	-		1.03 (0.66–1.62)	ns	0.97 (0.61–1.53)	ns	1.34 (0.86–2.07)	ns	1.22 (0.80–1.87)	ns
Q6 (bonds with other local people)	-		1.83 (1.26–2.68)	**	2.73 (1.83–4.07)	***	2.49 (1.63–3.81)	***	2.77 (1.75–4.38)	***

**Table 4:** The associations between disaster preparedness (A1) and factors (Model 2). CI = confidence interval. ref. = reference. EOAs = evacuation order areas. ns = *p* > 0.05, * *p* ≤ 0.05, ** *p* ≤ 0.01, *** *p* ≤ 0.001.

	2011		2012		2013		2014		2015	
	Odds Ratio (95% CI)		Odds Ratio (95% CI)		Odds Ratio (95% CI)		Odds Ratio (95% CI)		Odds Ratio (95% CI)	
Model 2										
women (ref)	1		1		1		1		1	
men	0.67 (0.48–0.91)	*	0.64 (0.46–0.88)	**	0.63 (0.45–0.87)	**	0.88 (0.60–1.28)	ns	0.77 (0.54–1.09)	ns
20–29	1		1		1		1		1	
30–39	1.29 (0.67–2.50)	ns	2.00 (0.92–4.36)	ns	2.11 (0.88–5.04)	ns	1.35 (0.56–3.25)	ns	0.60 (0.26–1.38)	ns
40–49	1.03 (0.54–1.97)	ns	2.11 (0.95–4.66)	ns	2.97 (1.28–6.87)	*	1.59 (0.67–3.78)	ns	0.53 (0.23–1.19)	ns
50–59	1.22 (0.66–2.25)	ns	1.30 (0.60–2.80)	ns	2.09 (0.92–4.77)	ns	1.09 (0.46–2.58)	ns	0.75 (0.34–1.63)	ns
60 or over	1.09 (0.61–1.95)	ns	1.56 (0.76–3.19)	ns	2.64 (1.22–5.69)	*	2.08 (0.94–4.59)	ns	0.67 (0.33–1.36)	ns
mountainous area (ref)	1		1		1		1		1	
central area	1.57 (0.98–2.52)	ns	1.05 (0.67–1.66)	ns	1.32 (0.81–2.15)	ns	2.08 (1.16–3.74)	*	1.71 (0.98–2.97)	ns
coastal area other than EOAs	3.23 (1.85–5.65)	***	1.77 (1.03–3.04)	*	2.01 (1.14–3.56)	*	4.27 (2.20–8.30)	***	2.50 (1.33–4.67)	**
EOAs	1.78 (0.83–3.82)	ns	1.44 (0.68–3.06)	ns	1.46 (0.64–3.35)	ns	2.74 (1.13–6.64)	*	2.44 (0.99–5.98)	ns
Q1 (relief regarding regional disasters)	2.16 (1.53–3.04)	***	1.78 (1.27–2.49)	***	1.17 (0.83–1.65)	ns	0.85 (0.58–1.24)	ns	1.64 (1.14–2.35)	**

**Table 5:** The associations between disaster preparedness (A1) and factors (Model 3). CI = confidence interval. ref. = reference. EOA = evacuation order areas. ns = *p* > 0.05, * *p* ≤ 0.05, ** *p* ≤ 0.01, *** *p* ≤ 0.001.

	2011		2012		2013		2014		2015	
	Odds Ratio (95% CI)		Odds Ratio (95% CI)		Odds Ratio (95% CI)		Odds Ratio (95% CI)		Odds Ratio (95% CI)	
Model 3										
women (ref)	-		1		1		1		1	
men	-		0.68 (0.49–0.94)	*	0.74 (0.53–1.04)	ns	0.86 (0.59–1.24)	ns	0.84 (0.58–1.21)	ns
20–29	-		1		1		1		1	
30–39	-		1.98 (0.90–4.38)	ns	2.14 (0.88–5.20)	ns	1.29 (0.54–3.08)	ns	0.72 (0.30–1.72)	ns
40–49	-		2.19 (0.98–4.89)	ns	3.04 (1.29–7.18)	*	1.44 (0.62–3.37)	ns	0.52 (0.23–1.22)	ns
50–59	-		1.36 (0.63–2.97)	ns	2.05 (0.88–4.77)	ns	0.99 (0.43–2.29)	ns	0.77 (0.35–1.73)	ns
60 or over	-		1.57 (0.76–3.22)	ns	2.51 (1.14–5.50)	*	1.80 (0.85–3.79)	ns	0.61 (0.29–1.27)	ns
mountainous area (ref)	-		1		1		1		1	
central area	-		1.06 (0.65–1.72)	ns	1.19 (0.71–2.01)	ns	1.94 (1.09–3.44)	*	1.70 (0.94–3.08)	ns
coastal area other than EOAs	-		1.81 (1.04–3.17)	*	1.71 (0.93–3.15)	ns	4.31 (2.24–8.29)	***	3.15 (1.61–6.15)	***
EOAs	-		1.53 (0.70–3.36)	ns	1.41 (0.59–3.35)	ns	2.64 (1.10–6.32)	*	2.78 (1.08–7.17)	*
Q1 (relief regarding regional disasters)	-		1.58 (1.09–2.27)	*	1.05 (0.73–1.52)	ns	0.81 (0.56–1.19)	ns	1.40 (0.95–2.06)	ns
Q2 (relief regarding of radiation)	-		0.85 (0.58–1.25)	ns	0.94 (0.63–1.39)	ns	1.45 (0.97–2.17)	ns	0.93 (0.63–1.38)	ns
Q3 (familiarity with the medical service)	-		0.89 (0.57–1.38)	ns	1.40 (0.88–2.22)	ns	1.19 (0.73–1.97)	ns	1.46 (0.81–2.64)	ns
Q4 (familiarity with the welfare service)	-		1.37 (0.93–2.00)	ns	0.97 (0.64–1.46)	ns	1.48 (0.95–2.29)	ns	1.50 (0.93–2.42)	ns
Q5 (evaluation of prefectural reconstruction)	-		1.09 (0.71–1.68)	ns	0.90 (0.58–1.41)	ns	1.37 (0.89–2.11)	ns	1.19 (0.80–1.77)	ns
Q6 (bonds with other local people)	-		1.82 (1.27–2.59)	**	2.52 (1.74–3.67)	***	2.80 (1.85–4.22)	***	1.94 (1.30–2.90)	**

**Table 6:** The associations between health checkups (A2) and factors (Model 4). CI = confidence interval. ref. = reference. EOAs = evacuation order areas. ns = *p* > 0.05, * *p* ≤ 0.05, ** *p* ≤ 0.01, *** *p* ≤ 0.001.

	2011		2012		2013		2014		2015	
	Odds Ratio (95% CI)		Odds Ratio (95% CI)		Odds Ratio (95% CI)		Odds Ratio (95% CI)		Odds Ratio (95% CI)	
Model 4										
women (ref)	-		1		1		1		1	
men	-		0.99 (0.68–1.46)	ns	1.08 (0.71–1.64)	ns	1.57 (1.03–2.39)	*	1.21 (0.81–1.81)	ns
20–29	-		1		1		1		1	
30–39	-		1.73 (0.81–3.67)	ns	1.19 (0.54–2.63)	ns	2.12 (0.97–4.65)	ns	2.24 (0.98–5.15)	ns
40–49	-		1.82 (0.84–3.94)	ns	2.30 (1.02–5.19)	*	2.56 (1.16–5.66)	*	2.62 (1.17–5.89)	*
50–59	-		2.53 (1.19–5.34)	*	2.80 (1.26–6.23)	*	3.41 (1.55–7.51)	**	2.55 (1.16–5.61)	*
60 or over	-		3.39 (1.70–6.77)	***	2.76 (1.36–5.61)	**	3.80 (1.91–7.55)	***	4.02 (1.96–8.25)	***
mountainous area (ref)	-		1		1		1		1	
central area	-		0.69 (0.38–1.23)	ns	0.57 (0.28–1.16)	ns	0.85 (0.45–1.59)	ns	0.60 (0.32–1.13)	ns
coastal area other than EOAs	-		0.90 (0.45–1.78)	ns	0.57 (0.25–1.28)	ns	0.90 (0.44–1.84)	ns	0.57 (0.28–1.16)	ns
EOAs	-		0.90 (0.34–2.36)	ns	0.89 (0.29–2.80)	ns	0.98 (0.38–2.52)	ns	0.75 (0.26–2.17)	ns
Q1 (relief regarding regional disasters)	-		1.02 (0.66–1.58)	ns	0.95 (0.61–1.48)	ns	1.02 (0.67–1.55)	ns	1.10 (0.73–1.65)	ns
Q2 (relief regarding of radiation)	-		0.70 (0.44–1.10)	ns	1.53 (0.92–2.56)	ns	1.35 (0.85–2.15)	ns	1.06 (0.69–1.61)	ns
Q3 (familiarity with the medical service)	-		1.10 (0.67–1.80)	ns	1.76 (1.06–2.91)	*	1.16 (0.70–1.92)	ns	1.52 (0.88–2.63)	ns
Q4 (familiarity with the welfare service)	-		1.49 (0.97–2.28)	ns	1.40 (0.87–2.26)	ns	1.92 (1.21–3.06)	**	1.18 (0.72–1.94)	ns
Q5 (evaluation of prefectural reconstruction)	-		1.36 (0.78–2.36)	ns	1.00 (0.55–1.82)	ns	0.68 (0.41–1.11)	ns	0.80 (0.52–1.23)	ns
Q6 (bonds with other local people)	-		0.93 (0.62–1.39)	ns	1.94 (1.26–3.00)	**	1.09 (0.71–1.66)	ns	1.46 (0.97–2.21)	ns

**Table 7:** The associations between health checkups (A2) and factors (Model 5, under 50). CI = confidence interval. ref. = reference. EOAs = evacuation order areas. ns = *p* > 0.05, * *p* ≤ 0.05, ** *p* ≤ 0.01.

	2011		2012		2013		2014		2015	
	Odds Ratio (95% CI)		Odds Ratio (95% CI)		Odds Ratio (95% CI)		Odds Ratio (95% CI)		Odds Ratio (95% CI)	
Model 5 (under 50)										
women (ref)	-		1		1		1		1	
men	-		1.02 (0.57–1.83)	ns	0.93 (0.48–1.81)	ns	1.50 (0.76–3.00)	ns	1.39 (0.73–2.62)	ns
mountainous area (ref)	-		1		1		1		1	
central area	-		1.25 (0.55–2.88)	ns	1.15 (0.44–3.01)	ns	0.42 (0.13–1.41)	ns	0.54 (0.18–1.65)	ns
coastal area other than EOAs	-		2.15 (0.74–6.26)	ns	1.32 (0.41–4.30)	ns	0.37 (0.10–1.40)	ns	0.34 (0.10–1.16)	ns
EOAs	-		4.15 (0.74–23.34)	ns	4.37 (0.69–27.72)	ns	0.55 (0.11–2.62)	ns	0.26 (0.05–1.37)	ns
Q1 (relief regarding regional disasters)	-		1.73 (0.83–3.62)	ns	0.87 (0.44–1.72)	ns	1.50 (0.78–2.91)	ns	0.74 (0.38–1.44)	ns
Q2 (relief regarding of radiation)	-		1.01 (0.48–2.11)	ns	1.10 (0.49–2.47)	ns	0.96 (0.45–2.05)	ns	0.84 (0.42–1.69)	ns
Q3 (familiarity with the medical service)	-		1.27 (0.63–2.56)	ns	2.45 (1.15–5.25)	*	1.75 (0.81–3.75)	ns	1.28 (0.56–2.90)	ns
Q4 (familiarity with the welfare service)	-		1.63 (0.86–3.11)	ns	2.76 (1.28–5.91)	**	2.30 (1.12–4.71)	*	2.01 (0.93–4.33)	ns
Q5 (evaluation of prefectural reconstruction)	-		0.83 (0.38–1.82)	ns	0.77 (0.31–1.92)	ns	0.59 (0.27–1.32)	ns	0.74 (0.37–1.51)	ns
Q6 (bonds with other local people)	-		0.57 (0.30–1.06)	ns	1.57 (0.79–3.11)	ns	1.61 (0.84–3.08)	ns	1.69 (0.87–3.30)	ns

**Table 8:** The associations between health checkups (A2) and factors (Model 5, 50 or over). CI = confidence interval. ref. = reference. EOAs = evacuation order areas. ns = *p* > 0.05, * *p* ≤ 0.05.

	2011		2012		2013		2014		2015	
	Odds Ratio (95% CI)		Odds Ratio (95% CI)		Odds Ratio (95% CI)		Odds Ratio (95% CI)		Odds Ratio (95% CI)	
Model 5 (50 or over)										
women (ref)	-		1		1		1		1	
men	-		0.94 (0.56–1.60)	ns	1.25 (0.71–2.19)	ns	1.52 (0.88–2.62)	ns	1.12 (0.67–1.88)	ns
mountainous area (ref)	-		1		1		1		1	
central area	-		0.42 (0.17–1.02)	ns	0.10 (0.01–0.79)	*	1.19 (0.55–2.54)	ns	0.62 (0.29–1.35)	ns
coastal area other than EOAs	-		0.49 (0.18–1.31)	ns	0.10 (0.01–0.83)	*	1.34 (0.55–3.29)	ns	0.79 (0.32–1.92)	ns
EOAs	-		0.31 (0.09–1.07)	ns	0.11 (0.01–1.06)	ns	1.35 (0.37–4.96)	ns	1.83 (0.35–9.50)	ns
Q1 (relief regarding regional disasters)	-		0.73 (0.41–1.29)	ns	0.99 (0.55–1.78)	ns	0.80 (0.46–1.42)	ns	1.50 (0.89–2.50)	ns
Q2 (relief regarding of radiation)	-		0.50 (0.27–0.91)	*	1.91 (0.95–3.84)	ns	1.84 (1.00–3.40)	ns	1.07 (0.62–1.84)	ns
Q3 (familiarity with the medical service)	-		1.22 (0.58–2.53)	ns	1.13 (0.55–2.30)	ns	0.79 (0.39–1.59)	ns	1.85 (0.87–3.94)	ns
Q4 (familiarity with the welfare service)	-		1.41 (0.78–2.55)	ns	0.93 (0.49–1.77)	ns	1.80 (0.97–3.35)	ns	0.75 (0.38–1.49)	ns
Q5 (evaluation of prefectural reconstruction)	-		2.27 (0.99–5.21)	ns	1.18 (0.51–2.71)	ns	0.72 (0.38–1.36)	ns	0.84 (0.49–1.46)	ns
Q6 (bonds with other local people)	-		1.31 (0.76–2.27)	ns	2.02 (1.12–3.64)	*	0.85 (0.48–1.50)	ns	1.45 (0.85–2.47)	ns

## Supplementary Materials

The following are available online at http://www.mdpi.com/1660-4601/15/3/516/s1.
