# Supplementary material for: Social Capital Enhanced Disaster Preparedness and Health Consultations after the 2011 Great East Japan Earthquake and Nuclear Power Station Accident
Source: Int J Environ Res Public Health. 2018 Mar 14;15(3):516. doi: 10.3390/ijerph15030516 (PMC5877061; doi:10.3390/ijerph15030516)
Supplement: Supplementary file 1 [file ijerph-15-00516-s001.pdf]

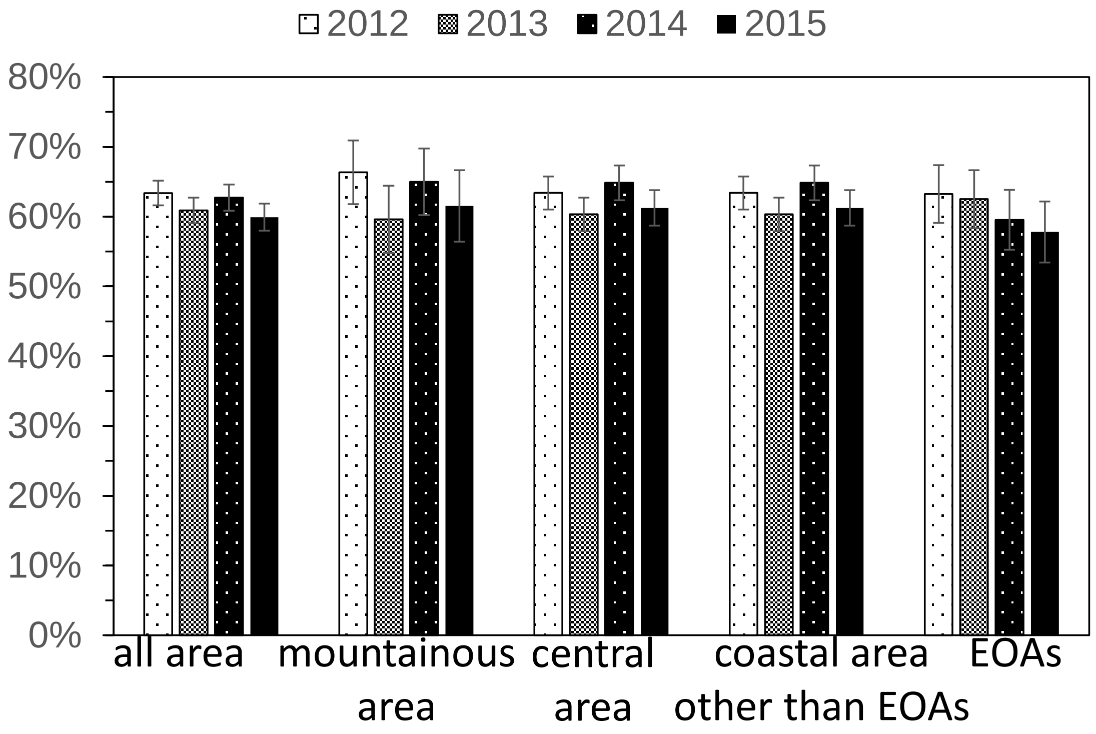

Figure. S-1. The yearly change in the percentage of people who experienced bonds with other local people. Error bar represents standard error. EOA = evacuation order areas.
